# Analysis of the Mechanical Properties and Damage Mechanism of Carbon Fiber/Epoxy Composites under UV Aging

**DOI:** 10.3390/ma15082919

**Published:** 2022-04-16

**Authors:** Zhongmeng Shi, Chao Zou, Feiyu Zhou, Jianping Zhao

**Affiliations:** 1 School of Mechanical and Power Engineering, Nanjing Tech University, Nanjing 211816, China; 15222390631@163.com (Z.S.); xinnongchaoer@gmail.com (C.Z.); pzhzfy123@163.com (F.Z.); 2 Jiangsu Key Lab of Design and Manufacture of Extreme Pressure Equipment, Nanjing 211816, China

**Keywords:** CFRP, ultraviolet aging, mechanical properties, nanoindentation, aging mechanisms

## Abstract

The UV durability of carbon fiber composites has been a concern. In this work, UV irradiation on carbon fiber-reinforced polymer (CFRP) materials was performed using an artificial accelerated UV aging chamber to investigate the effect of UV exposure on carbon fiber composites. UV aging caused some of the macromolecular chains on the surface resin to break, resulting in the loss of small molecules and loss of mass. After 80 days of UV irradiation exposure, a significant decline in the macroscopic mechanical properties occurred in the longitudinal direction, with the largest decrease of 23% in longitudinal compressive strength and a decreasing trend in the transverse mechanical properties at the later stage of aging. The microscopic mechanical properties of the CFRP specimens were characterized using nanoindentation, and it was found that UV aging had an embrittlement effect on the matrix, and its hardness/modulus values were higher than the initial values with UV exposure. The fibers were less affected by UV irradiation.

## 1. Introduction

Carbon fiber-reinforced plastics (CFRPs) are a series of high-performance composites made of carbon fiber as reinforcement and resin as matrix, using advanced composite molding and processing process. They have high strength-to-weight ratio and stiffness-to-weight ratio, acid and corrosion resistance, fatigue resistance, and designability. Due to the above characteristics, they are widely used in aerospace, rail transportation, pressure vessels, civil engineering, and other fields [1]. Due to the wide variety of applications, CFRPs are inevitably subjected to various environmental conditions such as UV irradiation, humidity, temperature, and load [2]. The matrix material of CFRPs is a polymer material, and since the birth of polymer material, it cannot avoid the problem of environmental aging; therefore, it is very meaningful to study UV irradiation, which significantly deteriorates the performance of CFRPs [3].

Polymeric materials undergo a “photo-oxidation reaction” when exposed to UV light [4]. The photo-oxidation reaction causes changes in the mechanical properties of polymeric materials due to the cross-linking and chain-breaking of macromolecular chains [5,6], resulting in changes in the glass transition temperature of the materials. It has been shown that the glass transition temperature (Tg) of fiber composites decrease slightly when exposed to UV irradiation [7]. Nicola Zavatta et al. performed thermal aging of carbon fiber/epoxy composites and found that for aging temperatures below Tg, a moderate decrease in strength was observed, and when aging temperatures were above Tg, a rapid decrease in strength was observed. This was attributed to the degradation of the epoxy matrix and the fiber/epoxy interface [8].

UV aging usually results in degradation of the properties of composites [9], although it has also been reported that UV aging produces a slight improvement in the mechanical properties of the carbon fiber/epoxy composites due to the post-curing. In a study reported in the literature [10], the improved tensile and compressive properties of fiber/epoxy composites in the early stages of aging were attributed to post-curing effects, and with increasing UV irradiation time, the macromolecular chains broke and the matrix material deteriorated leading to poor fiber–matrix adhesion. In another study [7], the carbon fiber/epoxy composites showed a slight increase in interlaminar shear strength under alternating UV/condensation cycle aging, possibly due to the decrease in residual stresses from curing.

It has been shown that the thickness of the degradation layer formed by resin and other polymer materials due to the fact of UV irradiation is approximately 300–500 µm [11,12], while the material beneath the degradation layer is affected to a limited extent. Therefore, it is of significance to study the mechanical properties of the aged surface, and with the development of nanoindentation techniques, it is possible to allow for the characterization of the surface mechanical properties of materials. M. Pecora et al. [13] performed nanoindentation tests on PR250 epoxy resin and its 3D interlock carbon fiber-reinforced composites before and after thermal aging, and the results showed that the elastic and plastic behavior was affected by thermal aging, while the viscoelastic behavior was not. Libin K. et al. [14] performed nanoindentation tests on carbon fiber/epoxy composites before and after UV aging and found that UV aging caused an increase in the modulus near the fibers, which was caused by high cross-linking. Most nanoindentation characterizations for UV aging of fiber composites have focused on the matrix perspective, without a more in-depth investigation of the reinforcing phase and the interfacial phase.

The main objective of this paper was to investigate the macroscopic/microscopic mechanical properties and aging mechanism of CFRPs by UV irradiation. At present, the macroscopic mechanical properties are mostly concentrated on one or several of the strength parameters of the laminate. This paper systematically compares the changes in multiple static mechanical properties of CFRP laminates (i.e., longitudinal/transverse tension, longitudinal compression, longitudinal bending, and in-plane and out-plane shear) with UV aging time. The UV aging micromechanical properties of CFRP phases (i.e., fiber, matrix, and interface) were investigated by the nanoindentation technique, and the UV irradiation aging mechanism of CFRPs was also explained from SEM, Tg, and weight loss perspectives.

## 2. Experiment

### 2.1. Materials

The fiber type used in this paper was Toray T700 carbon fiber, and the resin was bisphenol A-type epoxy resin. The hardener was dicyandiamide, according to a 10:1 mixing ratio, and the resin system curing temperature was 90–130 °C. More fiber and resin performance parameters are shown in Table 1 and Table 2.

### 2.2. CFRP Composite Preparation

**Figure 1:**
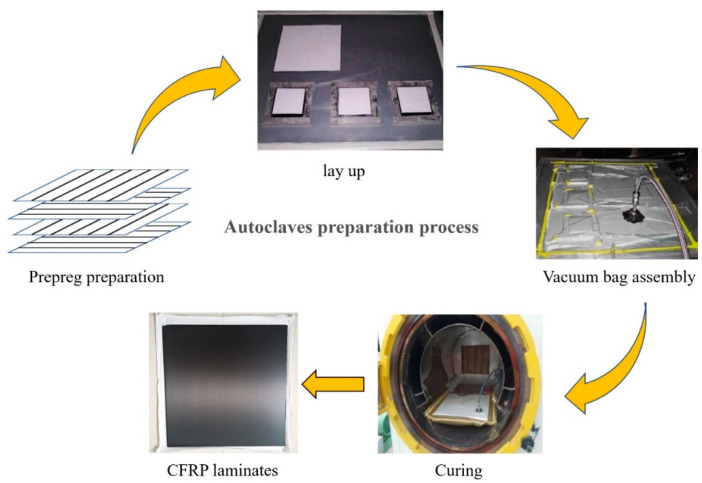
CFRP composite preparation process.

**Figure 2:**
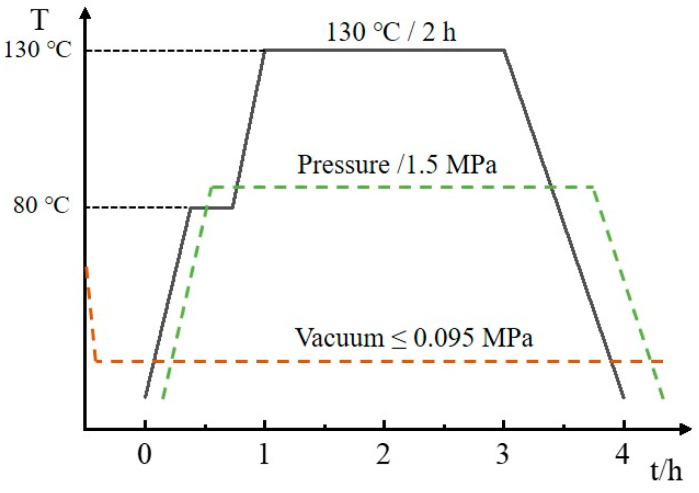
Autoclave molding process parameters.

The prepared 1 mm CFRP laminate lay-ups were [0_8_], and 2 mm laminates were [0/90]_4S_, where the fiber content was 60%. CFRP laminates were made using an autoclave molding process, and the preparation procedures are shown in Figure 1. First, the laminate lay-up design was considered, and the prepreg was cut to the desired shape. Then, the lay-up was performed, encapsulated in a vacuum bag, and placed in an autoclave for curing, where the curing temperature of the autoclave was 130 °C, the curing time was 2 h, and the pressure was 1.5 MPa, as shown in Figure 2. After the CFRP composites were cured, we waited for the temperature inside the tank to drop to room temperature and ensured complete pressure relief before opening the hatch to remove the material. Next, the raw edges of the composites were removed and numbered for backup. Finally, the required test specimens were cut.

### 2.3. Ultraviolet Aging Test

**Figure 3:**
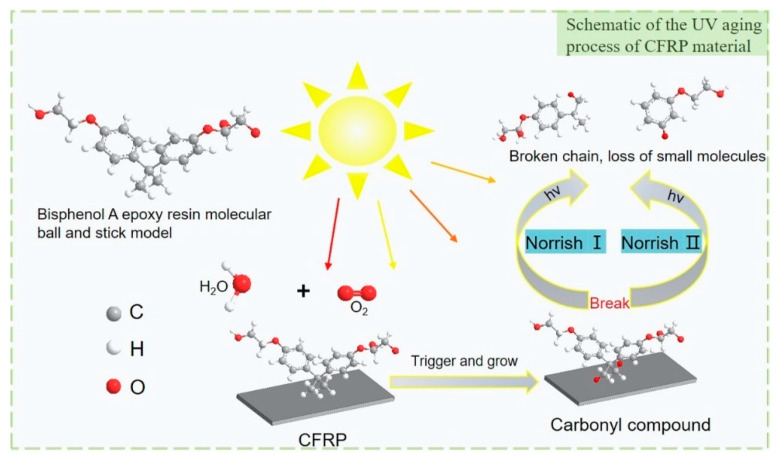
UV aging of CFRP material.

The main mechanism of CFRP thermal oxidation and photooxidative aging is the free radical reaction mechanism, which includes the chain trigger, chain growth, and chain-breaking stages. As shown in Figure 3, carbon fiber composites combine with water molecules and oxygen in the environment under UV irradiation to form oxidation products containing carbon radicals. As aging continues, cross-linking, branching, and molecular chain scission occur. These reactions will cause the fiber and matrix to de-bond, thus affecting the mechanical properties of the material [15].

**Figure 4:**
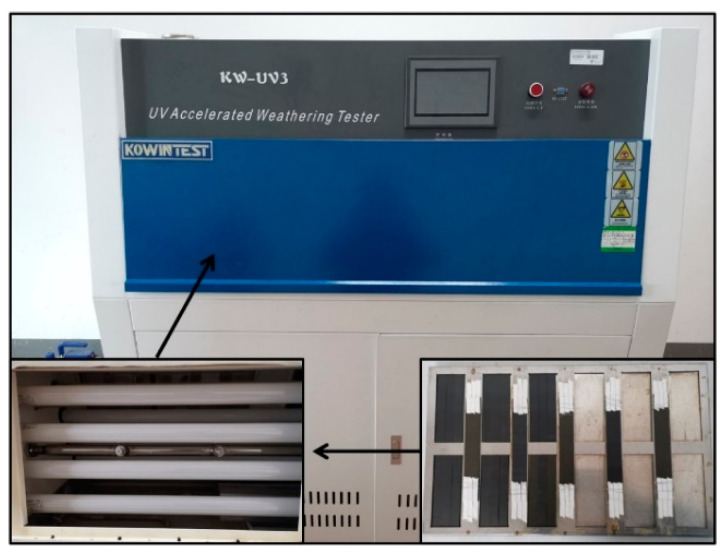
UV exposure chamber.

An artificial indoor UV irradiation accelerated aging test method was used to study the evolution of the mechanical properties of the carbon fiber composites under UV single factor without setting condensation conditions. The experimental apparatus adopted the KW-UV3-A UV-aging tester. As shown in Figure 4, four UVA-340 lamps placed at an angle emitted UV light at a wavelength of approximately 340 nm to ensure that each part of the specimen received the same amount of UV radiation. The temperature was set to 70 °C, and the irradiation intensity was set to 1 W/(m^2^·nm) according to the maximum power of the machine. Specimens were exposed to only one surface during irradiation and were periodically removed from the aging chamber for test characterization.

### 2.4. Macroscopic Mechanical Test

#### 2.4.1. Unidirectional Tensile and Compression Tests

Carbon fiber composites are anisotropic materials, and their longitudinal mechanical properties are much higher than their transverse mechanical properties. They have very high specific strength and stiffness. According to this characteristic, they are often used in service environments of long-term tensile load or compressive load; therefore, it is necessary to study its unidirectional tensile and compressive mechanical properties under UV irradiation.

**Figure 5:**
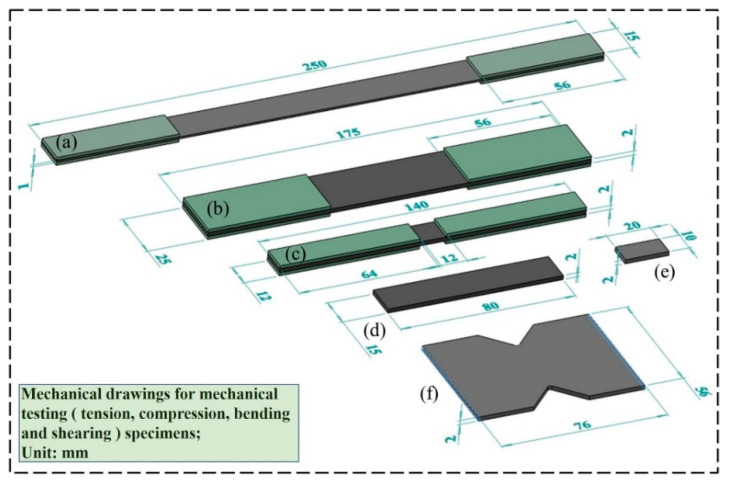
(**a**) 0° tensile specimen; (**b**) 90° tensile specimen; (**c**) 0° compression specimen; (**d**) bending specimen; (**e**) interlaminar shear specimen; (**f**) V-notched rail shear specimen.

The tensile mechanical properties of specimens were tested under different UV irradiation times. The tensile tests were conducted on an MTS 809 mechanical testing machine according to ASTM D3039 [16], and the specimens were cut into rectangular specimens of 250 × 15 × 1 mm^3^ in the 0° direction and 175 × 25 × 2 mm^3^ in the 90° direction according to the standard, as shown in Figure 5a,b. To avoid damage to the specimen caused by the fixture in the 0° tensile process and the discontinuity of the tensile load, glass fiber-reinforcement sheets were fixed on both ends of the specimen, which were similar to the modulus of CFRP and had good deformation coordination, and the size of the reinforcement sheets was 56 × 15 × 1.5 mm^3^. The choice of bonding agent between the carbon fiber specimen and the reinforcement sheet is crucial and will largely determine whether the ultimate tensile strength of the material can be tested. The specimens and the reinforcement sheets were glued with K-801-modified acrylate AB adhesive. The longitudinal tensile (0°) speed was 2 mm/min, and the transverse tensile (90°) speed was 1 mm/min. Five specimens were tested in each group, and the tensile strength and modulus obtained from the tests were recorded as the final test value by taking the average value.

Carbon fiber composites are often used in environments subjected to compressive loads. To investigate the effect of UV irradiation on the mechanical properties of CFRPs in compression, a unidirectional compressive specimen was designed according to ASTM D6641 [17]. The specimens were cut into 140 × 12 × 2 mm^3^ rectangular specimens, as shown in Figure 5c, and the reinforcement sheets’ size was 64 × 12 × 1.5 mm^3^. The compression tests were performed on an INSTRON 3382 tester, using a combined loading method (end and shear loading method) for CFRP tests. The compression rate was 1 mm/min. Five specimens were tested in each group, and the compressive strength and modulus obtained from the tests were recorded as the final test value by taking the average value.

#### 2.4.2. Flexural and Short Beam Shear Test 

To investigate the changes in the bending properties of the CFRPs during UV irradiation, a unidirectional flexural specimen was designed according to GB/T 3356-2014, and the flexural test was completed on an INSTRON 3382 material testing machine, According to the standard, the specimen was cut into a rectangular specimen of 80 × 15 × 2 mm^3^ (0° direction) as shown in Figure 5d. A three-point bend loading method was adopted, and the loading speed was 5 mm/min, the span-thickness ratio was 32:1, and the radii of the loading indenter and support were 5 mm. The irradiated surface was the one in compression (top) during the test. Five specimens were tested in each group, and the bending strength and modulus obtained from the test were recorded as the final test value by taking the average value.

Due to the unique structural characteristics of composite laminates, the interlaminar mechanical properties are lower than the intralaminar mechanical properties, and the bonding force between the layers deteriorates under the service environment of UV which, in turn, affects the overall mechanical properties. Therefore, it is extremely important to focus on the interlaminar shear mechanics of carbon fiber composites under the UV aging condition.

The interlaminar shear strength of CFRP laminates was tested by the short beam method according to ISO 14130 [18]. The specimens were cut into rectangular specimens of 20 × 10 × 2 mm^3^, as shown in Figure 5e, the loading speed was 1 mm/min, the radius of the rounded corner of the loading indenter was 3 mm, the radius of the rounded corner of the support was 2 mm, the three-point bending loading method was used, and the span–thickness ratio was 5:1. The irradiated surface was the one in compression (top) during the test. For each group of five specimens tested, the average interlaminar shear strength value was recorded as the final test value.

#### 2.4.3. V-Notched Rail Shear Test

**Figure 6:**
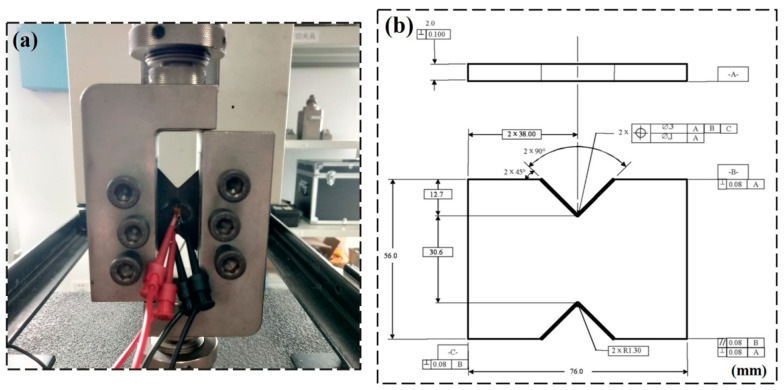
(**a**) V-notched rail shear apparatus illustration; (**b**) V-notched rail shear specimen mechanical drawing; all dimensions in mm.

The in-plane shear mechanical properties of CFRP laminates were tested using the V-notched rail shear test method, which has a higher accuracy compared to the traditional ±45° off-axis tension. The V-notched rail shear method was a combination product of the double rail shear method and Iosipescu shear method, using two pairs of loading rails to hold the V-notched specimen on both sides, when the tensile load was applied, the rails transferred the shear load to the specimen through the specimen surface as shown in Figure 6a. Compared with the Iosipescu shear method, this method uses in-plane shear loading instead of applying the load at the upper and lower ends of the specimen and uses a larger test cross-sectional area, which can provide a higher shear load on the specimen. The standard based on this method is ASTM D7078 [19], the specimen size is shown in Figure 6b, the loading rate was 2 mm/min, the specimen lay-up was [0/90]_4S_, and the specimen thickness was 2 mm. Strain gauges were pasted in the center of the specimen, and the strain gauges were located at ±45° of the loading axis at both ends of the specimen notch center. Five specimens were tested in each group, and the average value of the shear strength/modulus obtained from the test was recorded as the final test value.

### 2.5. Micromechanical Test

#### 2.5.1. SEM and Weightlessness Analysis

To investigate the UV aging mechanism of carbon fiber composites, the surface morphology of the CFRP specimens after UV irradiation was investigated by scanning electron microscopy (SEM). The specimens were cut into small pieces (10 × 10 × 1 mm^3^), treated with gold spraying, and fixed with conductive double-sided tape. 

To investigate the effect of UV exposure on the weight of CFRP materials, an electronic balance with a weighing accuracy of 0.1 mg was used for weighing, with a specimen size of 155 × 10 × 1 mm^3^ and an initial weight of 3.7612 g. The mass stabilized during the later stages of aging, so the test was followed for only 45 days.

#### 2.5.2. FTIR Analysis

The changes in functional groups of composite samples before and after UV aging were studied using an FTIR spectrometer (Nicolet Model 6700) to investigate the aging mechanism. The scanning range was from 4000 to 500 cm^−1^ with a resolution of 4 cm^−1^.

#### 2.5.3. DMA Analysis

To investigate the thermomechanical properties of CFRP materials, the specimens were tested with a dynamic thermomechanical analyzer (DMA-1) before and after UV aging, and the specimens were cut to 30 ×10 × 1 mm^3^ for unidirectional lay-up with reference to ISO-6721 [20]. The loading method was three-point bending, the span–thickness ratio was 20, the vibration frequency was 1 HZ, the heating rate was 5 °C/min, and the heating range was 20–210 °C.

#### 2.5.4. Nanoindentation Test

**Figure 7:**
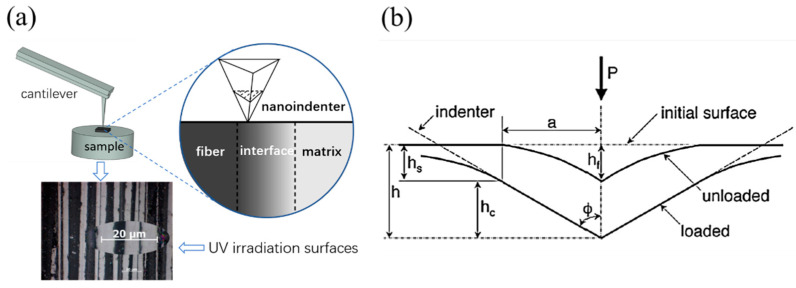
Illustrative descriptions of (**a**) a schematic diagram of nanoindentation test; (**b**) schematic plot of nanoindentation parameters.

**Figure 8:**
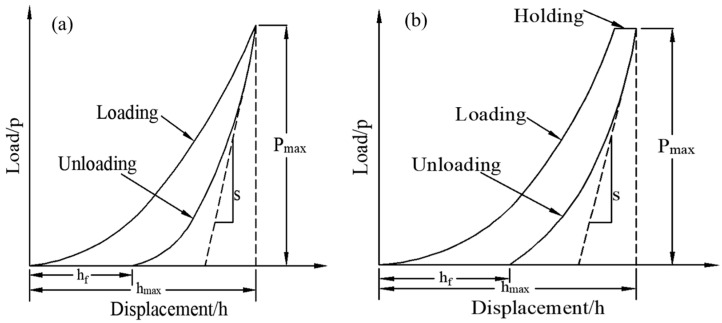
Typical load–displacement curve for nanoindentation: (**a**) curve with no load holding; (**b**) curve with load holding.

Nanoindentation technology is based on the elastic contact theory, which was proposed by Oliver and Pharr in 1992 [21]. The mechanical properties of tiny regions of materials can be studied at the nanoscale. This paper used nanoindentation to test the effect of UV aging on the micromechanics (i.e., hardness/modulus) of each phase of the carbon fiber composites (Figure 7). The test surface was the surface of the CFRP specimen (15 × 15 × 1 mm^3^) before and after UV irradiation, and the loading mode was the force-controlled mode of 20-5-20 (20 s loading, 5 s holding, 20 s unloading) with a maximum load of 5 mN (fiber and interface zones) and 1 mN (matrix zone). The hardness H and the elastic modulus (E) can be obtained from load–displacement curves such as those illustrated in Figure 8, where *P*_max_ is the peak indentation load, *h*_max_ is the maximum press-in depth, h_f_ is the residual indentation depth, and S is the unloading stiffness. The hardness is given by [22]:

(1) $$H=\frac{P_{\max}}{A_{c}}$$

where $\;A_{c}$ is the contact area and $h_{c}$ is the contact depth as is shown in Figure 7b. When using Berkovich indenter, the contact area, $A_{c}$, is a function of $h_{c}$ [23]:

(2) $$A_{c}=F(h_{c})=Dh_{c}^{2}+D_{1}hc+D_{2}h_{c}^{\frac{1}{2}}+D_{3}h_{c}^{\frac{1}{4}}+D_{4}h_{c}^{\frac{1}{8}}+D_{5}h_{c}^{\frac{1}{16}}$$

where the value of *D* is 24.56; *D*_1_, *D*_2_, *D*_3_, *D*_4_, and *D*_5_ are fitting parameters for nonideal head contacted areas such as Berkovich indenters.

(3) $$h_{c}=h_{\max}-\varepsilon \frac{P_{\max}}{S}$$

where ε is a constant that depends on the type of the indenter. ε=0.75 for Berkovich indenters.

The reduced modulus ($E_{r}$) can be obtained from the unloading curve (Figure 8) and is calculated as follows [24]:

(4) $$E_{r}=\frac{S\sqrt{\pi }}{2\sqrt{A_{c}}}=\frac{\sqrt{\pi }}{2\sqrt{A_{c}}}\frac{dp}{dh}$$

where $S=\frac{dp}{dh}$ is the unloading contact stiffness; $A_{c}$ is the contact area.

The elastic modulus *E* can be calculated from the following equation:

(5) $$\frac{1}{E_{r}}=\frac{1-\nu^{2}}{E}+\frac{1-\nu_{in}^{2}}{E_{in}}$$

where ν is the Poisson ratio of the specimen material; $E_{in}$ and $\nu_{in}$ are the elastic modulus and Poisson’s ratio of the indenter material, respectively.

## 3. Results and Discussion

### 3.1. Weightlessness and Surface Morphology Analysis

**Figure 9:**
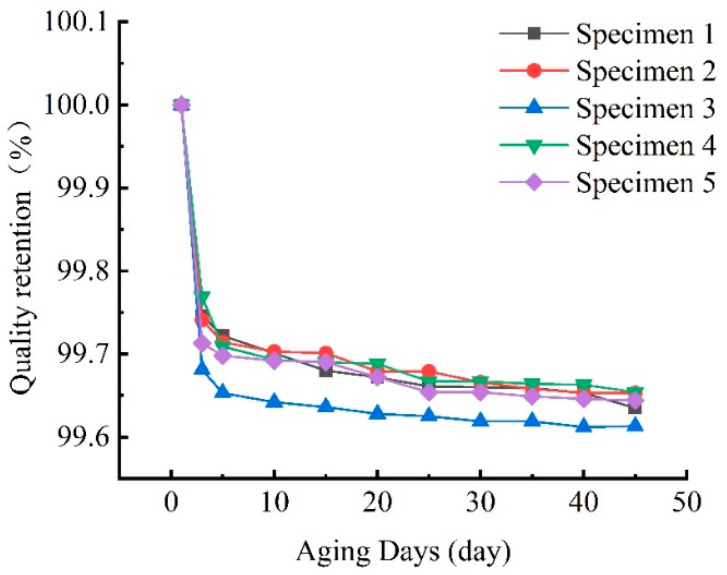
The relationship curve between mass retention rate and time under UV irradiation.

As can be seen in Figure 9, the mass loss was faster in the initial stage of UV irradiation, mainly due to the volatilization of water molecules caused by high temperature, and then became slower in the late stage of aging, mainly due to the formation of surface resin shedding, which was also confirmed in the SEM microscopic morphology below. As the aging time increases, the mass loss will become quite limited.

**Figure 10:**
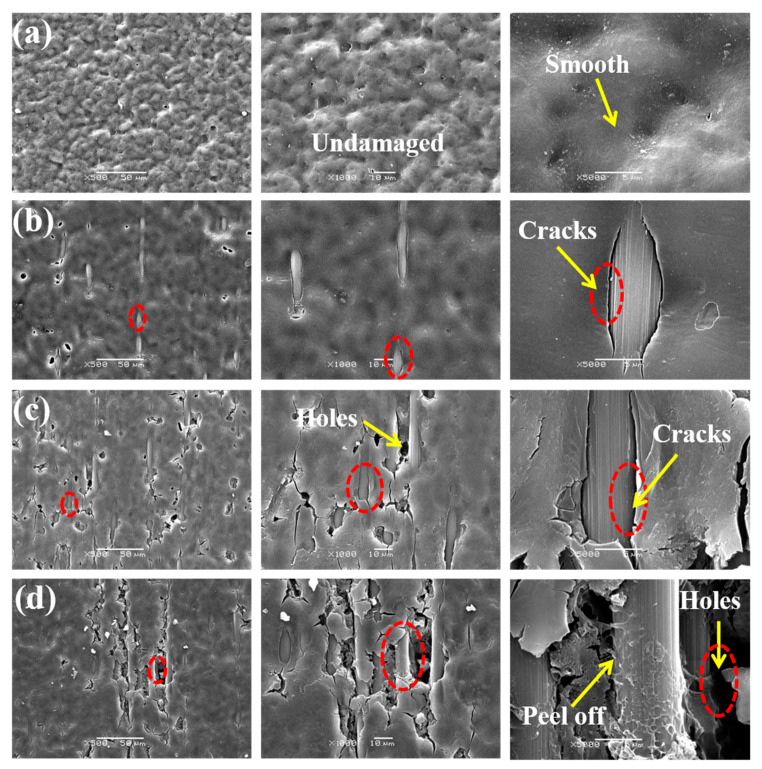
Surface microscopic morphology of CFRP specimens with different UV aging times: (**a**) unaged; (**b**) 10 days; (**c**) 40 days; (**d**) 80 days.

With the increase of UV irradiation time, the damage on the surface of the specimen gradually increased, as shown in Figure 10, and the surface of the unaged specimen was uniformly covered with resin matrix, no fiber exposure, and after 10 days of UV aging, the phenomenon of fiber exposure was produced, fine cracks were created at the junction of the fibers with the matrix, and no obvious damage occurred to the matrix. After 40 days of aging, there was more obvious damage to the matrix, many holes appeared on the surface of the specimen, and the gap where the fibers bond to the matrix increased. The matrix on the surface of the specimen also produced cracks, and the damage further increased. After 80 days of aging, the holes were further enlarged, and some fibers were completely exposed, the cracks in the matrix began to expand inward and the resin matrix was peeled off, which explained the loss of quality of the specimens in the late aging period.

UV aging had an obvious damage effect on carbon fiber composites, the main mechanism is as follows: ultraviolet rays are more energetic and active compared with visible light, and the energy of UV can make some of the covalent bonds break. Therefore, under UV irradiation, resin and other macromolecular chains break, and the small molecular fragments produced by the break will occupy a large amount of space and produce small cracks. Moreover, resin and fiber have different coefficients of thermal expansion, the deformation of the resin is greater than that of the fiber at high temperatures, which generates thermal stress at the interface and destroys the deformation coordination relationship between fiber and resin, causing the fiber and resin to de-bond, further aggravating the expansion of cracks.

### 3.2. Fourier Transform Infrared Spectroscopy (FTIR) Analysis

**Figure 11:**
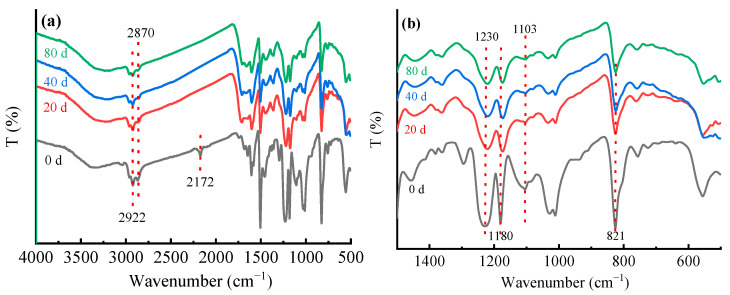
The FTIR spectra of the CFRP before and after aging: (**a**) full spectrum; (**b**) fingerprint region.

As shown in Figure 11, the absorption peak at 2172 cm^−1^ disappeared after 20 days of UV irradiation, which may be the C≡C/C≡N stretching vibration peak in the epoxy resin curing agent dicyandiamide, after UV aging, all the unreacted curing agents remaining in the epoxy resin participated in the reaction, so the characteristic peak disappeared, which also indirectly proves that UV aging will make the composites produce post-curing. The absorption peaks at 2922 cm^−1^ and 2870 cm^−1^, related to C-H, decreased and the susceptible hydrocarbon groups were oxidized on the surface of the CFRP specimens after a long period of aging. The small shoulder peak near 1750–1716 cm^−1^ was a carbonyl absorption peak resulting from chain-break degradation of the epoxy resin. In addition, the absorption peaks of aromatic ether (1230 cm^−1^), aliphatic ether (1103 cm^−1^), 1180 cm^−1^ (C-O), and C-H absorption peak of disubstituted benzene near 821 cm^−1^ decreased, indicating that the UV aging process caused some of the chemical bonds to oxidize, chain-break, and generate free reactive radicals, and the reactive radicals combined to generate small molecules and become loose, which was the direct cause of the resin peeling off of the surface layer, macroscopically manifested as weight loss.

### 3.3. Dynamic Thermomechanical Analysis (DMA)

**Figure 12:**
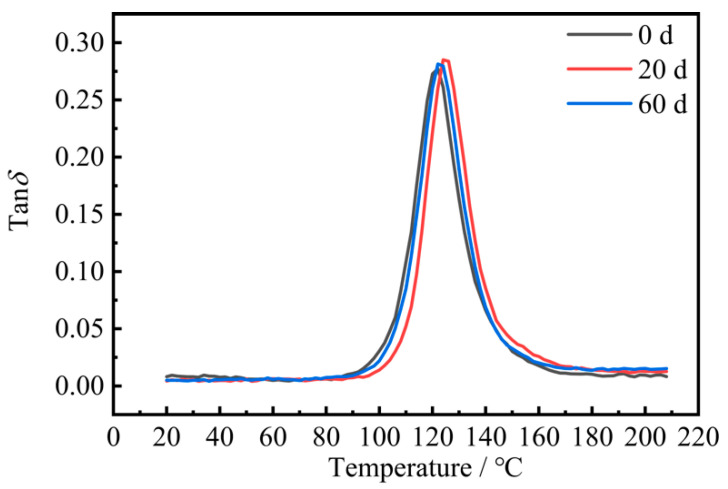
CFRP loss factor–temperature curve.

Figure 12 shows the loss factor curves of the CFRP composites before and after UV aging, and the peak temperature of the loss factor was the glass transition temperature (Tg) of the composites. The glass transition temperature (Tg) was 121 °C before aging, Tg was 125 °C after 20 days of aging, and Tg decreased to 122 °C after 60 days of aging, with the glass transition temperature first increasing and then decreasing.

In the early stage of UV aging, UV irradiation produced post-curing of the composites and cross-linking reaction, which made the glass transition temperature increase. In the late stage of UV aging, in the epoxy resin group under UV irradiation, chain-breaking occurred, cross-link density decreased, and the damage effect of UV was greater than the favorable effect of the post-curing part, resulting in a decrease in the glass transition temperature and a decrease in the thermal stability of the material.

### 3.4. Tensile Tests Results and Discussion

**Figure 13:**
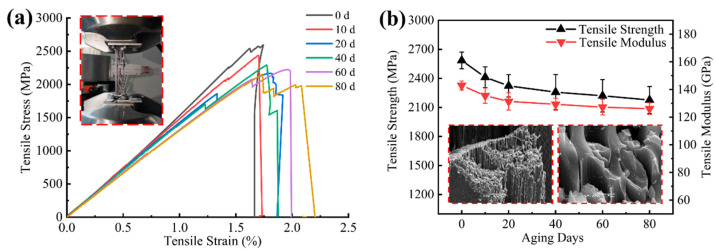
(**a**) Longitudinal direction tensile stress-strain curve; (**b**) longitudinal tensile strength/modulus versus aging time.

The longitudinal tensile stress–strain curve showed typical brittle fracture characteristics, and the tensile strength and modulus decreased with the increase in UV aging time as shown in Figure 13. The strength decreased by 16% and the modulus decreased by 12% after 80 days of aging. The decline rate was faster in the first 20 days of UV aging and tended to level off in the later ultraviolet exposure stage, which was due to the fact that UV aging of carbon fiber composites mainly acts on the surface layer, and it is difficult for the damage to continue to expand to the interior as the aging time increases. UV aging reduced the degree of bonding between fibers and resin, and although the fibers were less affected by UV irradiation, the overall load-bearing capacity of the composite depends on the degree of bonding between the reinforcement phase and the matrix phase, and the debonding of the surface layer fibers from the matrix will lead to premature failure of some fibers during tension. As shown in Figure 13a, the tensile process of the specimens in the later aging stage showed premature fracture of some fibers, which made the integral stress–strain curves produce a stepped shape.

**Figure 14:**
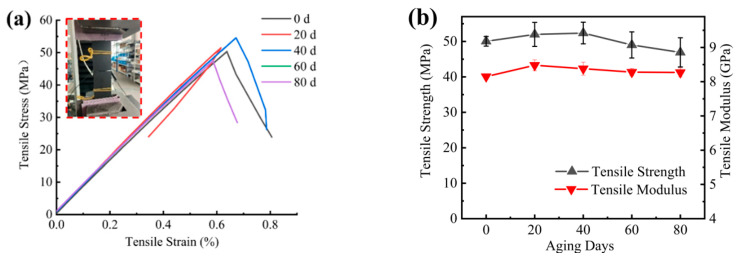
(**a**) Transversal direction tensile stress–strain curve; (**b**) transversal direction tensile strength/modulus versus aging time curve.

The 90° tensile specimen corresponded to the mechanical properties of the matrix. As shown in Figure 14, the transverse tensile strength/modulus tended to increase at the beginning of aging and then decreased at the later stage of aging as the post-curing effect was weaker than the UV aging effect [25], which is in accordance with the aging law of polymer materials [26,27,28]. The strength decreased by 6%, and the modulus increased by 1% after 80 days of aging. The highest increase in modulus was 4% after 20 days of aging. The highest increase in strength was 5% after 40 days of aging. The transverse tensile strain at break decreased by 7% after 80 days of aging, indicating that UV aging made the matrix brittle.

### 3.5. Compression Tests Results and Discussion

**Figure 15:**
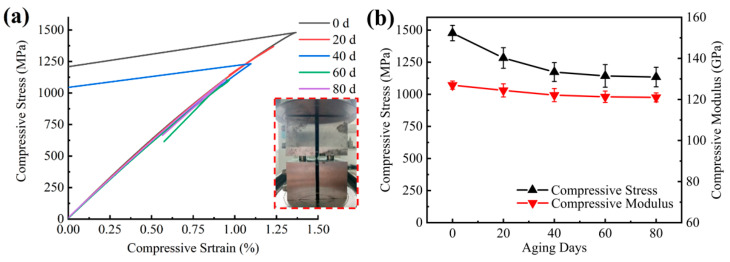
(**a**) Longitudinal direction compression stress–strain curve; (**b**) longitudinal direction compression strength/modulus versus UV aging time curve.

Longitudinal compression and longitudinal tension had similar mechanical property change trends with aging time as shown in Figure 15. The decline was faster in the early aging period and tended to level off in the latter aging period, and its strength decreased by 23% and the modulus decreased by 5% over 80 days of aging. The damage mode mostly presented as blooming in the middle of the gauge (BGM) and various longitudinal splits of the gauge (SGV), which is an acceptable failure mode, and the experimental data were valid.

### 3.6. Flexural Tests and Interlaminar Shear Tests Results and Discussion

**Figure 16:**
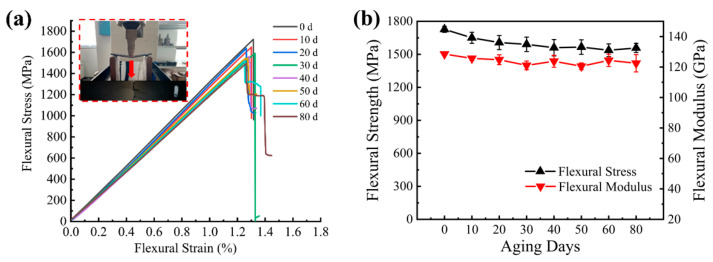
(**a**) Longitudinal direction flexural stress–strain curve; (**b**) longitudinal direction flexural strength/modulus versus UV aging time curve.

Bending and interlaminar shear tests were conducted on CFRP using the three-point bending method. The main failure damage mode of three-point bending was compression failure, since the compression failure strain (1.3%) of the material was less than the tensile failure strain (1.7%). Therefore, the failure of the upper surface occurred first as shown in Figure 16a. The flexural strength/modulus decreased rapidly in the first 20 days of UV aging, and a fluctuation in the decline trend was seen in the later aging period, with the flexural strength decreasing by 10% and the flexural modulus decreasing by 5% after 80 days of UV as shown in Figure 16b. The damaging effect of UV irradiation on the surface layer of the specimen leads to poor bonding between the fiber and the matrix, which is the cause of its flexural mechanical decline.

**Figure 17:**
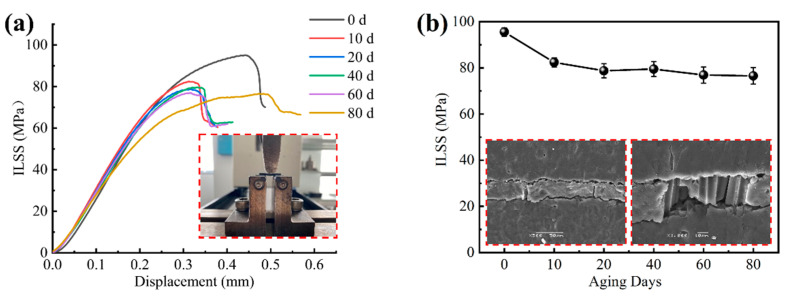
(**a**) Interlaminar stress–displacement curve; (**b**) interlaminar shear strength versus UV aging time curve.

The interlaminar shear strength decreased faster in the first 10 days of UV aging, and the decreasing rate tended to level off in the later ultraviolet exposure stage as shown in Figure 17. With the increase in loading force, the layer on the layer produced out-of-plane shear stress, which caused the damage between the layers and transferred it to the surface of the specimen; when the maximum interlaminar load of the specimen was reached, the surface of the specimen then produced a crack, as shown in Figure 17b, and the fibers partially fractured. After 80 days of aging, the interlaminar shear strength decreased by 20%. UV aging has a more pronounced effect on the interlaminar properties of CFRP laminates. The interlaminar shear strength reflects the overall bonding strength of the composite and is an important evaluation index of the mechanical properties of fiber composites.

### 3.7. V-Notched Rail Shear Test Results and Discussion

**Figure 18:**
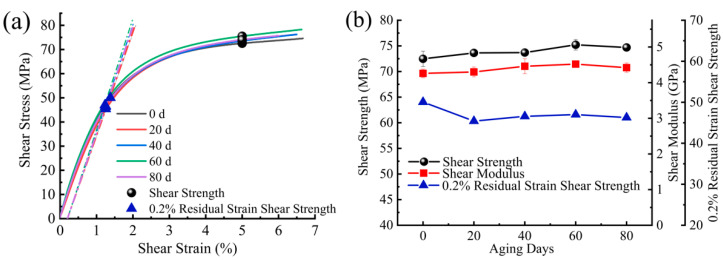
(**a**) V-notched shear stress–strain curve (in-plane); (**b**) in-plane shear properties during exposure to UV radiation.

The in-plane shear strength/modulus showed an increasing trend at the beginning of aging as shown in Figure 18. This may be because the in-plane shear mechanical properties were more sensitive to the post-curing effect between the fibers and the resin and showed an overall upward trend. The value of 0.2% strain is usually used in engineering as the tolerance value for setting the residual strain, and the corresponding residual strain shear strength was noted as $\tau_{s\left(0.2\right)}$. The residual strain shear strength was used to determine the approximate linear limit of the material stress–strain curve, starting with the residual strain tolerance value of 0.2% and making a straight line with the slope of the secant elastic modulus (shear modulus). The stress value at the intersection with the shear stress–strain curve was the residual strain shear strength. The residual strain shear strength showed an overall decreasing trend, with the greatest decrease at the beginning of aging, 9% at 20 days of aging, and 8% at 80 days of aging.

### 3.8. Nanoindentation Test Results and Discussion

The macroscopic mechanical properties above are a characterization of the overall mechanical properties of the carbon fiber composites under UV aging, and their overall macroscopic mechanical properties are closely related to the mechanical properties of the components. Therefore, it is necessary to study the micromechanical behavior of each component under ultraviolet aging at the micro- and nanoscale.

**Figure 19:**
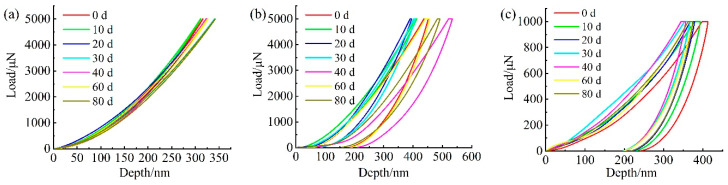
Nanoindentation test load vs. indentation depth curve at different aging times: (**a**) fiber phase; (**b**) interface phase; (**c**) matrix phase.

**Figure 20:**
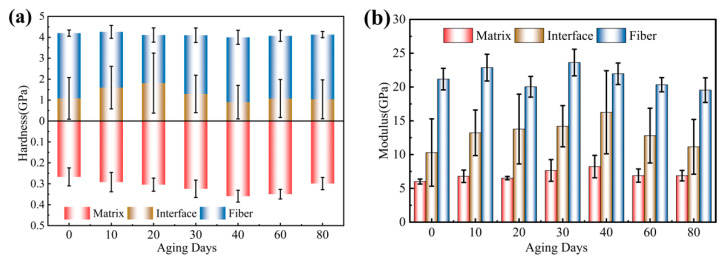
(**a**) Relationship between hardness and aging time for each component; (**b**) relationship between the modulus of each component and aging time.

Typical load displacements of the nanoindentation response at each phase of the UV-aged surface of the carbon fiber composite are shown in Figure 19. The elastic–plastic properties of the material can be analyzed from the perspective of the area-energy method enclosed by the indentation curve [29,30]. As shown in Figure 19, the area enclosed by the loading and unloading curves represents the energy loss during the indentation process, which is caused by plastic deformation, and the larger the area, the more plastic the material. It can be seen that the load–displacement curve of the fiber showed typical brittle material characteristics, as shown in Figure 19a, the matrix material showed typical plastic material characteristics, and the interface phase was in between with a certain plastic toughness. With the increase in UV exposure, the maximum indentation depth of each phase changed, and the indentation depth responded to the hardness characteristics of the material, while the unloading stiffness was related to the elastic modulus, according to Oliver and Pharr’s theory [21,22]. The microhardness and elastic modulus values that could be obtained from the indentation curve are shown in Figure 20.

The hardness of the fibers was less affected by UV irradiation, and the modulus had an increasing–decreasing trend, which may be due to the effect of UV irradiation on the graphite microcrystalline structure of the fibers, resulting in a decrease in their modulus at the later stage of aging, while the hardness and modulus of the matrix were more affected by UV irradiation, which had an embrittlement effect on the matrix material. The overall hardness and modulus tended to increase, and the modulus/hardness reached the maximum values after 40 days of aging, and the modulus increased by 37% and the hardness increased by 35%, respectively. The high hardness fibers will play a strengthening role on the surrounding low hardness resin. In the early aging period, the UV embrittlement effect of the resin at the interface was the dominant effect, leading to the increase in its hardness, and in the late aging period, the near-fiber effect declined, leading to the premature decrease in its hardness. The modulus of the interface area has a similar law to that of the matrix.

## 4. Further Discussion

UV irradiation resulted in cracking of the surface of the CFRP composites, generation of holes, and the deterioration of the bond between resin and fiber. It led to the deterioration of its mechanical properties. As shown in Table 3, transverse tensile and in-plane shear are non-principal directional mechanical properties of the material, which were less affected by the degree of bonding between fibers and resin, and their aging law is similar to that of polymers. Its strength/modulus increased at the beginning of aging, which was the result of the post-curing effect of the resin polymer material, and decreased as the exposure time increased, and the post-curing was weaker than the UV aging effect. The principal directional mechanical properties of the material (tensile, compression) depend on the degree of bonding between the fibers and the resin, and UV exposure breaks this bond, and the unfavorable part of UV irradiation is greater than its favorable part (post-curing), making its strength/modulus decrease with aging time.

**Figure 21:**
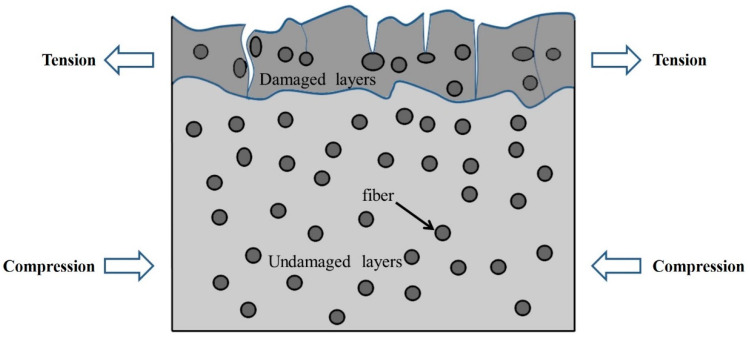
Schematic diagram of damaged layer crack expansion.

As UV aging acts only on the surface layer, the surface resin peels off causing a reduction in free volume, and the damaged surface layer tries to contract, but the undamaged layer at the bottom prevents it from contracting. This constraint introduces tensile stresses in the damaged layer and compressive stresses in the undamaged layer as shown in Figure 21 [11]. This tensile stress results in cracking of the damaged layer, which explains the further expansion of cracks on its surface. This leads to a continuous deterioration of the mechanical properties of the material.

The effect of UV aging on the mechanical properties of the CFRP materials was determined by the interaction of post-curing and UV degradation effects. The UV post-curing effect caused the molecular chains to cross-link, leading to an increase in material strength at the early stages of aging, while the degradation effect of UV light also caused a decrease in the strength of the fiber/matrix interface. This caused the mechanical properties that were more sensitive to the fiber/matrix bond strength (longitudinal tensile, longitudinal compression, interlaminar shear) to decrease more rapidly during UV irradiation. The mechanical properties that were more sensitive to post-curing were enhanced at the beginning of aging such as transverse tensile and V-notched shear.

The ultimate goal of accelerated aging is to predict material life. The main methods currently used to predict polymer matrix composites are the linear relationship method, Arrhenius model method, and remaining strength model method [31,32]. Among them, the remaining strength model is widely used to predict the life prediction of polymer matrix composites.

(6) $$S=S_{0}+\eta (1-e^{-\lambda t})-\beta \ln(1+\theta t)$$

The strength degradation of longitudinal tensile, longitudinal compression, bending, and interlaminar shear were more obvious under UV exposure, and the residual strengths under different irradiation times were fitted, and the fitting results are shown in Table 4.

The remaining strength under longer UV aging can be predicted by the fitted equation. After 800 days of UV aging, the longitudinal tensile residual strength was 1879 MPa (73%), longitudinal compression residual strength was 1879 MPa (51%), flexural residual strength was 1322 MPa (77%), and interlaminar shear residual strength was (71%). The compression strength decreased at the fastest rate and was sensitive to UV aging, which is the key point to focus on during the service of CFRP materials.

Further research will focus on UV aging at multiple temperature cycles to correlate natural UV aging with laboratory UV aging.

## 5. Conclusions

In this paper, the durability and damage mechanism of carbon fibre-reinforced resin matrix composites were investigated by accelerated UV aging treatment for 80 days. The conclusions are as follows.

(1) The UV aging had a deteriorating effect on multiple mechanical properties, with the most significant effect on the mechanical properties in the longitudinal direction, where the strength/modulus decreased with increasing UV exposure time. The longitudinal compression strength decreased the most by 23% due to the deterioration of the mechanical properties of the fiber/matrix interface caused by UV aging. The mechanical properties in the non-principal direction (transverse tensile, V-notched shear) increased in the early stage of UV aging, which was due to the post-curing effect, and caused the molecular chains to cross-link. A decreasing trend was also observed in the late aging period. This was due to the weaker post-curing effect than the degradation effect of UV irradiation in the latter stages of UV aging;
(2) According to FTIR analysis, the epoxy resin matrix was degraded under UV exposure, and the chemical bonds or molecular forces in the chain segments were broken, resulting in free reactive radicals, which reacted with oxygen in the air to form small molecules, such as peroxides, causing some of the epoxy resin to peel off and form fragments and holes. The SEM showed that some of the gaps between the fibers and the matrix increased and the bonding became worse. This was the reason why the mechanical properties of the CFRP were reduced;
(3) The nanoindentation results showed that the hardness of fibers was less affected by UV irradiation, while the hardness and modulus of the matrix were more affected by UV irradiation, which had a brittle effect on the matrix material. The overall hardness and modulus tended to increase. The high hardness fibers will play a strengthening role on the surrounding low hardness resin. In the early aging period, the UV embrittlement effect of the resin at the interface was the dominant effect, leading to the increase in its hardness, and in the late aging period, the near-fiber effect declined, leading to the premature decrease in its hardness. The interface region and the matrix have a similar law in terms of modulus;
(4) Through the remaining strength model, it can be concluded that after 800 days of aging, the compression remaining strength retention rate was only 51%, and it was more sensitive to the UV aging effect, which is the key point to pay attention to in the service process of CFRP materials.

## Figures and Tables

**Table 1:** T700 carbon fiber main parameters.

Fiber Type	Filaments	Tensile Strength (MPa)	Young’s Modulus (GPa)	Elongation (%)	GSM (g/m^2^)
T700	12 K	4827	234	2.1	193

**Table 2:** Resin system’s main parameters.

Epoxy Resin	Viscosity (Pa.s)	Epoxy Value (eq/100 g)	Volatile Components (%)
Y04	10–16	0.48–0.54	≤2

**Table 3:** Macromechanics of CFRP pre- and post-exposure.

Test Type		Retention of Strength and Modulus				
		10 Days	20 Days	40 Days	60 Days	80 Days
**Longitudinal Tension**	**S**	93%	90%	87%	85%	84%
	**E**	95%	92%	90%	89.%	88%
**Transverse Tension**	**S**	/	104%	105%	98%	94%
	**E**	/	104%	103%	102%	101%
**Longitudinal Compression**	**S**	/	87%	79%	77%	77%
	**E**	/	98%	96%	96%	95%
**Bending**	**S**	96%	93%	90%/	89%	90%
	**E**	98%	97%	96%	97%	95%
**ILSS**	S	86%	82%	83%	80%	80%
**V-Notched Shear**	**S**	/	102%	102%	104%	103%
	**E**	/	101%	105%	106%	104%

**Table 4:** Remaining strength model fitting results.

Test Type	Fitting Formula	R^2^
**Longitudinal Tension**	S=2585.11−128.43ln(1+0.303t)	0.91
**Longitudinal Compression**	S=1477−156.06ln(1+0.132t)	0.93
**Bending**	S=1727−82.56ln(1+0.167t)	0.95
**ILSS**	S=95.52−3.068ln(1+9.053t)	0.98

## Data Availability

Not applicable.
